# Adenomatous Polyposis Coli Mutation Leads to Myopia Development in Mice

**DOI:** 10.1371/journal.pone.0141144

**Published:** 2015-10-23

**Authors:** Zhen Liu, Fangfang Qiu, Jing Li, Zhenzhen Zhu, Wenzhao Yang, Xiangtian Zhou, Jianhong An, Furong Huang, Qiongsi Wang, Peter S. Reinach, Wei Li, Wensheng Chen, Zuguo Liu

**Affiliations:** 1 Eye Institute of Xiamen University, Fujian Provincial Key Laboratory of Ophthalmology and Visual Science, Xiamen, China; 2 School of Ophthalmology and Optometry and Eye Hospital, Wenzhou Medical University, Wenzhou, Zhejiang, China; 3 Zhejiang Provincial Key Laboratory of Ophthalmology and Optometry, Wenzhou, Zhejiang, China; Cedars-Sinai Medical Center; UCLA School of Medicine, UNITED STATES

## Abstract

Myopia incidence in China is rapidly becoming a very serious sight compromising problem in a large segment of the general population. Therefore, delineating the underlying mechanisms leading to myopia will markedly lessen the likelihood of other sight compromising complications. In this regard, there is some evidence that patients afflicted with familial adenomatous polyposis (FAP), havean adenomatous polyposis coli (APC) mutation and a higher incidence of myopia. To clarify this possible association, we determined whether the changes in pertinent biometric and biochemical parameters underlying postnatal refractive error development in APC^Min^ mice are relevant for gaining insight into the pathogenesis of this disease in humans. The refraction and biometrics in APC^Min^ mice and age-matched wild-type (WT) littermates between postnatal days P28 and P84 were examined with eccentric infrared photorefraction (EIR) and customized optical coherence tomography (OCT). Compared with WT littermates, the APC^Min^ mutated mice developed myopia (average -4.64 D) on P84 which was associated with increased vitreous chamber depth (VCD). Furthermore, retinal and scleral changes appear in these mice along with: 1) axial length shortening; 2) increased retinal cell proliferation; 3) and decreased tyrosine hydroxylase (TH) expression, the rate-limiting enzyme of DA synthesis. Scleral collagen fibril diameters became heterogeneous and irregularly organized in the APC^Min^ mice. Western blot analysis showed that scleral alpha-1 type I collagen (col1α1) expression also decreased whereas MMP2 and MMP9 mRNA expression was invariant. These results indicate that defective APC gene function promotes refractive error development. By characterizing in APC^Min^ mice ocular developmental changes, this approach provides novel insight into underlying pathophysiological mechanisms contributing to human myopia development.

## Introduction

Myopia is a global public health problem severely impacting on the quality of life [1]. Higher degrees of myopia are a risk factor for ocular complications, such as glaucoma, retinal degeneration, and choroidal neovascularization leading to permanent visual impairment and even blindness [2,3]. Although numerous potential human risk factors have been identified, the underlying mechanisms contributing to ocular growth regulation and refractive error development remain largely unclear.

Recent studies on myopia in humans and animal models suggest that excessive axial length elongation [4,5], increased retinal proliferation [6], degenerative scleral changes [7,8] are associated with myopia development. Although the molecular mechanisms underlying these changes require further elucidation, this condition alters expression levels of various neurotransmitter mediators, and hormones including muscarinic receptors [9]. Dopamine (DA) pharmacology [10] and responses induced by retinoic acid [11], glucagon [12] and EGR-1(also named ZENK) [13] which contribute to myopia and visual development are also modified by, DA control modulation affects ocular growth by acting mostly as a ‘stop signal’ of this process [14]. Such a role is indicated for DA since retinal DA levels and TH activity declined in eyes deprived of sharp vision by using either diffusers (form deprivation myopia, FDM) or minus lenses (lens induced myopia, LIM) [15–17].

The adenomatous polyposis coli (APC) gene has 15 exons, localized to the long arm of chromosome 5(5q21- q22) [18]. APC is an ubiquitously expressed tumor suppressor protein, which has essential roles in regulating cell cycle progression, migration, differentiation and apoptosis [19]. During early embryonic eye development, APC mRNA is abundantly expressed in the neural retinal and retinal pigment epithelial (RPE) cells [20]. The majority of sequence aberrations in APC are frameshift or nonsense mutations leading to truncated protein expression [21]. More than 50 adenomatous polyps in the colon and rectum [22] are characteristic of familial adenomatous polyposis (FAP) caused by APC mutations. Congenital retinal pigment epithelial hypertrophy (CHRPE) is the most frequent manifestation of FAP [22]. Several APC-mutant mouse models have been generated resembling the FAP and colon cancer phenotype [23]. Mice with a disrupted APC gene also develop RPE hypertrophy [24]. It has been reported that 10 out of 14 FAP individuals with refraction anomalies had myopia ranging from -0.5 to -10.0 diopters (mean, -3.1 diopters) [22]. These considerations are suggestive of a potential role for an APC mutation contributing to refractive error development.

In the present study, we examined in APC^Min^ mice the association between myopia development and time dependent changes in refractive and biometric parameters to elucidate the functional role of an APC mutation (APC^Min^) in this process. The APC^Min^ mice generated by random ethylnitrosourea (ENU) mutagenesis, carries in the APC gene a nonsense mutation at codon 850 leading to adult onset anemia and multiple intestinal neoplasia (Min) [25]. We observed a greater myopic shift, increased vitreous chamber depth and scleral collagen fibril rearrangement. In addition, retinal proliferation increased whereas TH expression declined.

## Materials and Methods

### Experimental Animals

Age-matched male APC^Min^ mice (stock number 002020) on the C57BL/6 background and wild-type (WT) male C57BL/6 mice (purchased from Nanjing Biomedical Research Institute of Nanjing University, China) were used for this study. All animals were housed in cages at 25°C, on a 12:12 light-dark hour cycle, with food and water available *ad libitum*. Luminance in the cages was approximately 200 lux. All procedures were performed in accordance with the ARVO Statement for the Use of Animals in Ophthalmic and Vision Research. The protocols in our study were approved by the Committee on the Ethics of Animal Experiments of Xiamen University (Permit Number: XMUMC2012-12-9)

APC^Min^ mice were genotyped following a PCR protocol recommended by the Jackson Laboratory. A total of 26 mice were separated into 13 APC^Min^ mice and 13 wild-type mice. Each group underwent a series of ocular measurements at each of the following 6 postnatal time points: 28, 35, 42, 56, 70 and 84 days. All samples and measurements were obtained during the light period, at least two hours after lights-on and two-hours before lights-off. First, their refractive state was measured. Subsequently, mice were anesthetized by intraperitoneal injection of 1.2% ketamine (70 mg/kg body weight) /1.6% xylazine (10 mg/kg body weight) mixture and ocular dimensions were measured.

### Biometric Measurements

Postnatal refractive state development of the right eyes was characterized based on measurements of corneal radius of curvature (CRC), pupil diameter (PD), ocular anterior chamber depth (ACD), lens thickness (LT), vitreous chamber depth (VCD) and axial length (AL) on 28, 35, 42, 56, 70 and 84 days. There was no systematic left vs right eye anisometropia. Briefly, refractive state and PD were measured in a darkened room with an eccentric infrared photorefractor (EIR)[26]. The mice were gently positioned in front of the photoretinoscope, and swiftly repositioned until a clear first Purkinje image appeared in the center of the pupil. The measured refractive errors were then recorded by using software designed by Schaeffel et al [27]. Measurements were repeated at least three times for each eye. The CRC was measured with a keratometer (OM-4; Topcon Corporation, Dongguan, Japan), modified by mounting a +20.0 diopter (D) aspherical lens [28]. Each eye was measured three times to obtain a mean value. A custom-made real-time OCT instrument measured the AL and other ocular parameters [29]. After being anesthetized, each mouse was observed using a video viewing system for final orientation and positioning. The ACD was defined as the distance from the posterior surface of the cornea to the anterior surface of the lens. The VCD was defined as the distance from the back of the lens to the nerve fiber layer of the retina. The AL was defined as the distance between the anterior surface of the cornea and the vitreous-retina interface. Finally, after evaluating the refractive indices for each component of the eye, they were used to convert the recorded optical path length into a geometric path length. Each eye was scanned three times.

### Transmission Electron Microscopy

APC^Min^ and wild type mice were sacrificed by cervical dislocation at P84. The right eyes of five wildtype and APC^Min^ mice were evaluated. The anterior segment of the eye including the cornea, iris, and crystalline lens was cut away from the anterior scleral rim, and the vitreous body and retina were also dissected and discarded, leaving only the sclera. Posterior scleral tissue was fixed in a mixture composed of 2.5% glutaraldehyde and 4% paraformaldehyde in PBS (pH = 7.4) for 2 h. Then, the scleras were cut into 1×1mm pieces for further fixing, embedding, slicing, staining, and examined with a transmission electron microscope (JEM2100HC; JEOL, Tokyo, Japan).

### Immunostaining

Cryostat sections (10 μm in thickness) of each eyeball were fixed in cold acetone, and blocked with 2% normal bovine serum for 1h at room temperature. Sections of central retina were incubated with primary antibodies for TH (Millipore, AB152, 1:1000) and Ki67 (Abcam, ab15580, 1:300) overnight at 4°C and washed thoroughly with PBS. After further incubation in FITC-conjugated IgG (Invitrogen, 1:300), sections were counterstained with DAPI (Vector, H-1200) mounted, and photographed using a confocal laser scanning microscope (Fluoview 1000, Olympus, Tokyo, Japan).

### Western Blot

Retinal and scleral homogenates were resolved by SDS-PAGE and then blotted with specific antibodies for Ki67 (Abcam, ab15580, 1:200), TH (Millipore, AB152, 1:1000) and col1α1 (Santa Cruz, sc-28657, 1:1000) overnight at 4°C. Detection was achieved with an anti-rabbit-horseradish peroxidase (RD, HAF008, 1:1000). The signal was detected with a chemiluminescence kit (ECL, Thermo, 32106). Each protein band was normalized to β-actin expression levels in the same gel.

### Quantitative Real-time Reverse Transcription (RT)-PCR

Total RNA of sclera was isolated using TRIzol reagent (Invitrogen, USA), and its purity was confirmed by the OD260/280 nm absorption ratio (>1.8). Total RNAs (1μg) was reverse transcribed to cDNA using a cDNA Synthesis Kit (Takara, China). Quantitative real-time RT- PCR (Q-RT-PCR) was performed with a StepOne Real-Time PCR detection system (Applied Biosystems, Carlsbad, CA, USA) using an SYBR Premix Ex Taq Kit (Takara, China) according to the manufacturer’s instructions. Q-RT-PCR was performed in a 20 μL reaction under the following conditions: 95°C for 10 min, followed by 40 cycles of amplification at 95°C for 10 s and 60°C for 30 s. All experiments were performed in triplicate. The specific gene products were amplified using the following primer pairs: β-actin, 5- agccatgtacgtagccatcc -3 and 5-ctctcagctgtggtggtgaa -3; col1α1, 5- gagagcgaggccttcccgga-3 and 5- gggagccagcgggaccttgt-3; MMP2, 5- gttcaacggtcgggaataca-3 and 5- gccatacttgccatccttct-3; MMP9, 5- gactacgataaggacggcaaat-3 and 5- agatgaacgggaacacacag -3. A non-template control was included to evaluate the level of DNA contamination. The mRNA levels were analyzed by the comparative Ct method and normalized using β-actin.

### Statistical Analysis

Data analysis was conducted with commercial software (SPSS, ver.13.0; SPSS, Chicago, IL and Prism 5). Differences between WT and APC^Min^ genotypes at indicated postnatal days were analyzed by the independent Student t-tests. The Spearman linear correlation was used to test for significant relationships between the refractive error and the other ocular parameters. The value of each parameter is reported as the mean ± SEM of the right eyes of 13 mice in each age group. Values of western blot and Q-RT-PCR are shown as mean ± SD. A value of *P* < 0.05 was considered statistically significant. Significance levels are denoted by asterisks (*P <0.05, **P <0.01, ***P <0.001; ns, not significant).

## Results

### Relative myopia development in APC^Min^ mice from P28 to P84

**Fig 1:**
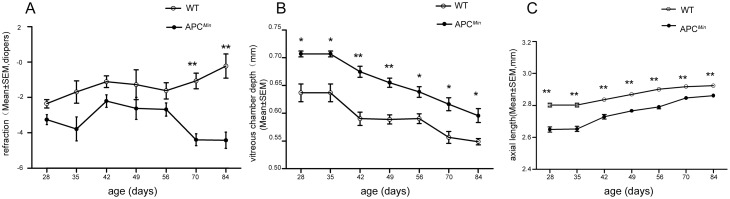
APC^Min^ mice have a greater myopic shift and longer vitreous chamber depth (VCD) than the WT littermates during postnatal development. Comparison of refractive status (A), VCD (B), and AL (C), between APC^Min^ mice and WT littermates at the indicated postnatal time points from P28 to P84. The asterisk denotes a significant difference between APC^Min^ mice and WT mice. *P <0.05, ** *P*<0.01, independent Student- t-tests, WT: n = 13, APC^Min^ mice: n = 13.

APC^Min^ mice seldom live longer than 140 days because they develop intestinal bleeding and severe anaemia [23]. This limitation accounts for why ocular dimensions in each of ten APC^Min^ mice and age-matched wildtype (WT) mice were only measured from P28 to P84 (Table 1). Consistent with previous reports [26], the refractive error of the WT mice, measured by EIR, increased rapidly in the hyperopic direction. Although APC^Min^ mice also developed myopic shifts before P56, there was no diopter difference between them and WT mice. Nevertheless, APC^Min^ mice were an average of -4.64D more myopic than the WT littermates on P84 (WT: n = 13, APC^Min^: n = 13, *P*<0.05, Fig 1A). To further characterize ocular growth in APC^Min^ mice, we measured AL and VCD with custom-built biometric equipment specifically designed for mice. VCD in both genotypes decreased with time, and there was a significant difference between the two genotypes after P28 (P<0.05) (Fig 1B). The VCD in APC^Min^ mice was longer than in WT littermates after P28 (e.g. 0.59 ± 0.04 mm VS 0.55 ± 0.01 mm at P84), which is consistent with VCD elongation observed in myopic human eyes [30]. AL in both genotypes increased during postnatal development. However, the AL in APC^Min^ mice was significantly shorter at each time point compared with that in WT mice (e.g. 2.86 ± 0.03 mm VS 2.92 ± 0.02 mm at P84; Fig 1C), which is inconsistent with the clinical features of myopia [30].

**Table 1:** Comparison of biometric parameters in WT and APC^Min^ mice.

Age (d)	Refraction (D)	PD (mm)	CRC (mm)	ACD (mm)	LT (mm)	RCALS (mm)	VCD (mm)	AL (mm)	BW (g)
P28									
WT	-2.35±0.23	2.03±0.07	1.34±0.03	0.39±0.01	1.70±0.06	1.67±0.17	0.63±0.06	2.80±0.01	10.90±0.66
Mt	-3.25±0.28	2.03±0.07	1.33±0.02	0.34±0.01**	1.50±0.04**	1.32±0.12	0.71±0.02*	2.65±0.01**	14.90±1.07*
P35									
WT	-1.69±0.63	2.14±0.08	1.33±0.02	0.39±0.02	1.66±0.06	1.16±0.05	0.63±0.06	2.80±0.01	16.70±0.66
Mt	-3.78±0.68	2.23±0.09	0.31±0.03	0.34±0.01**	1.50±0.05**	1.15±0.07	0.71±0.02*	2.65±0.01**	19.28±0.53*
P42									
WT	-1.11±0.33	2.16±0.08	1.33±0.02	0.41±0.01	1.72±0.04	1.15±0.06	0.59±0.03	2.84±0.01	20.07±0.43
Mt	-2.20±0.35	2.17±0.10	1.28±0.03	0.38±0.02**	1.58±0.04**	1.09±0.07	0.67±0.04**	2.73±0.01**	20.91±0.25
P49									
WT	-1.28±0.84	2.17±0.09	1.34±0.03	0.43±0.02	1.75±0.04	1.11±0.05	0.59±0.03	2.87±0.03	22.04±0.44
Mt	-2.62±0.62	2.24±0.07	1.27±0.02	0.40±0.01*	1.61±0.03**	1.09±0.06	0.65±0.03**	2.77±0.01**	21.70±0.27
P56									
WT	-1.63±0.46	2.27±0.07	1.39±0.02	0.43±0.01	1.76±0.03	1.20±0.09	0.59±0.02	2.90±0.01	23.73±0.39
Mt	-2.67±0.37	2.25±0.08	1.33±0.02	0.40±0.01*	1.66±0.03**	1.18±0.06	0.64±0.03*	2.79±0.01**	22.72±0.33
P70									
WT	-1.06±0.45	2.28±0.09	1.33±0.03	0.43±0.01	1.81±0.02	1.11±0.05	0.56±0.03	2.92±0.01	24.75±0.57
Mt	-4.39±0.34**	2.26±0.05	1.31±0.02	0.40±0.02	1.72±0.03**	1.13±0.05	0.62±0.03*	2.85±0.03**	23.67±0.25
P84									
WT	-0.22±0.68	2.30±0.07	1.36±0.02	0.43±0.02	1.82±0.03	1.19±0.07	0.55±0.01	2.92±0.02	27.87±0.55
Mt	-4.42±0.46**	2.32±0.07	1.36±0.03	0.42±0.01	1.73±0.05**	1.16±0.11	0.59±0.04*	2.86±0.01**	26.93±0.43

WT, wild type; Mt, APC^Min^ mice; PD, pupil diameter; CRC, corneal radius of curvature; ACD, anterior chamber depth; LT, lens thickness; RCALS, radius of curvature of anterior lens surface; VCD, vitreous chamber depth; AL, axial length; BW, body weight. Data are expressed as mean ± SEM (WT: n = 13, Mt: n = 13). Independent sample Student t-tests were applied between WT and APC^Min^ group.

* *P* < 0.05

** *P* < 0.01 compared with the WT group.

### Abnormal anterior segment growth in APC^Min^ Mice

**Fig 2:**
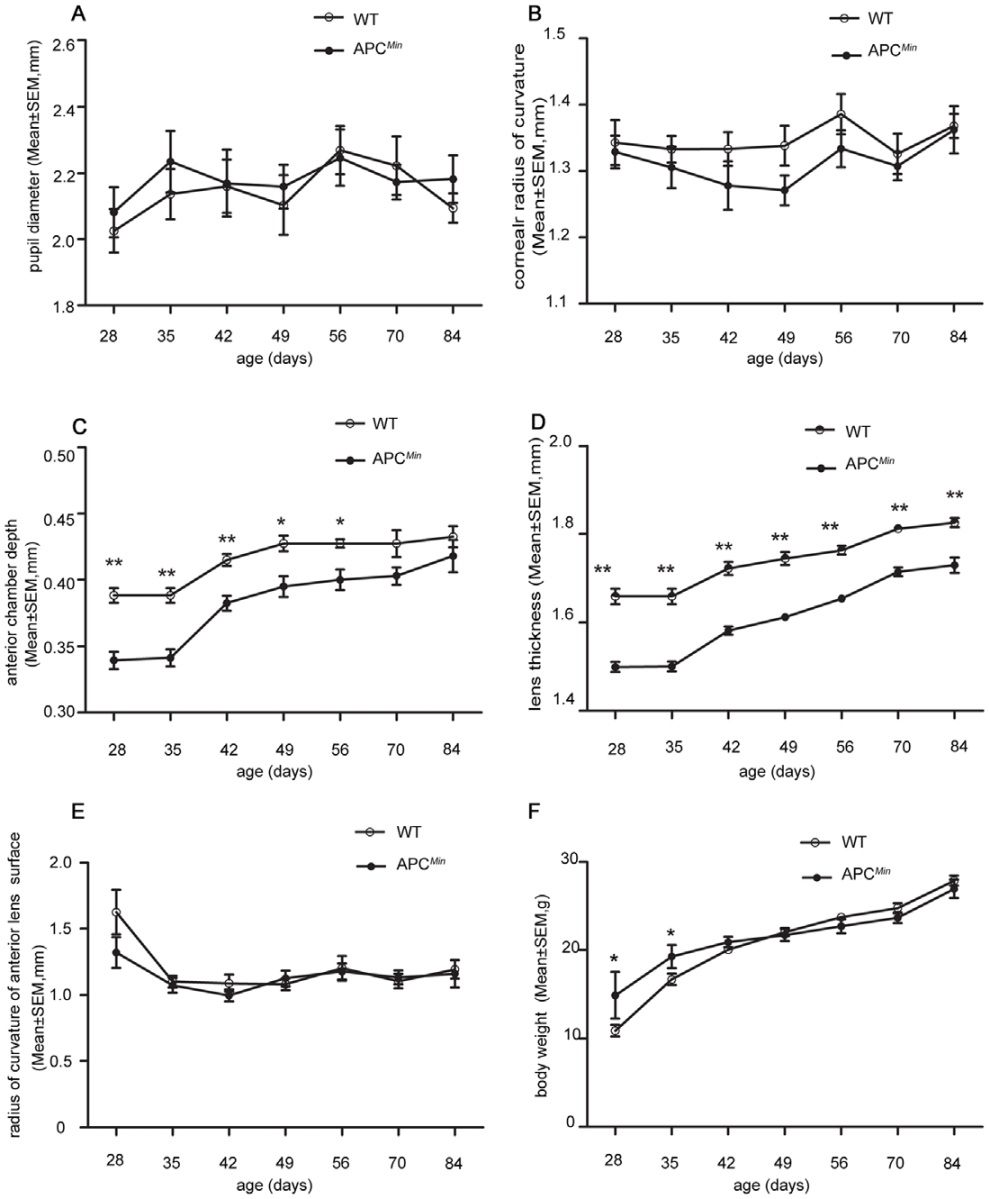
Comparison of changes in biometric parameters of APC^Min^ mice and WT littermates during postnatal development. APC^Min^ mice and WT littermates were evaluated for pupil diameter (PD) (A), radius of corneal curvature (CRC) (B), anterior chamber depth (ACD) (C), lens thickness (LT) (D), radius of curvature of anterior lens surface (RCALS) (E) and body weight (F) from P28 to P84. There was no difference in PL, CRC, and RCALS during this time period. However, ACD is shorter in APC^Min^ mice than in WT from P28 to P56; LT in APC^Min^ mice is smaller than in WT from P28 and P84; body weight in the APC^Min^ mice were heavier than the WT mice from P28 toP35. The asterisk denotes a significant difference between APC^Min^ mice and WT mice. *P <0.05, ** *P*<0.01, independent sample Student t-tests, WT: n = 13, APC^Min^ mice: n = 13.

Both PD and CRC increased during postnatal development, and there was no significant difference between the two genotypes at any time point (Fig 2A and 2B). Similarly, ACD and LT increased during postnatal development in both genotypes, whereas ACD in APC^Min^ mice was shorter than in the WT from P28 to P56. On the other hand, this difference did not persist after P70 (Fig 2C). Meanwhile, LT in APC^Min^ mice was significantly shorter than that in the WT mice (P<0.05), and the anterior lens surface radius of curvature of (RCALS) after P28 was the same in both groups (P>0.05) (Fig 2D and 2E).

To evaluate whether the development of myopia is confounded by a change in APC^Min^ mice body weight, we weighed them during postnatal development. Their weights progressively increased without any difference between the two genotypes after P42, but the APC^Min^ mice were heavier than the WT mice at P28 and P35 (Fig 2F).

### Retinal morphological and proliferative changes in APC^Min^ mice

**Fig 3:**
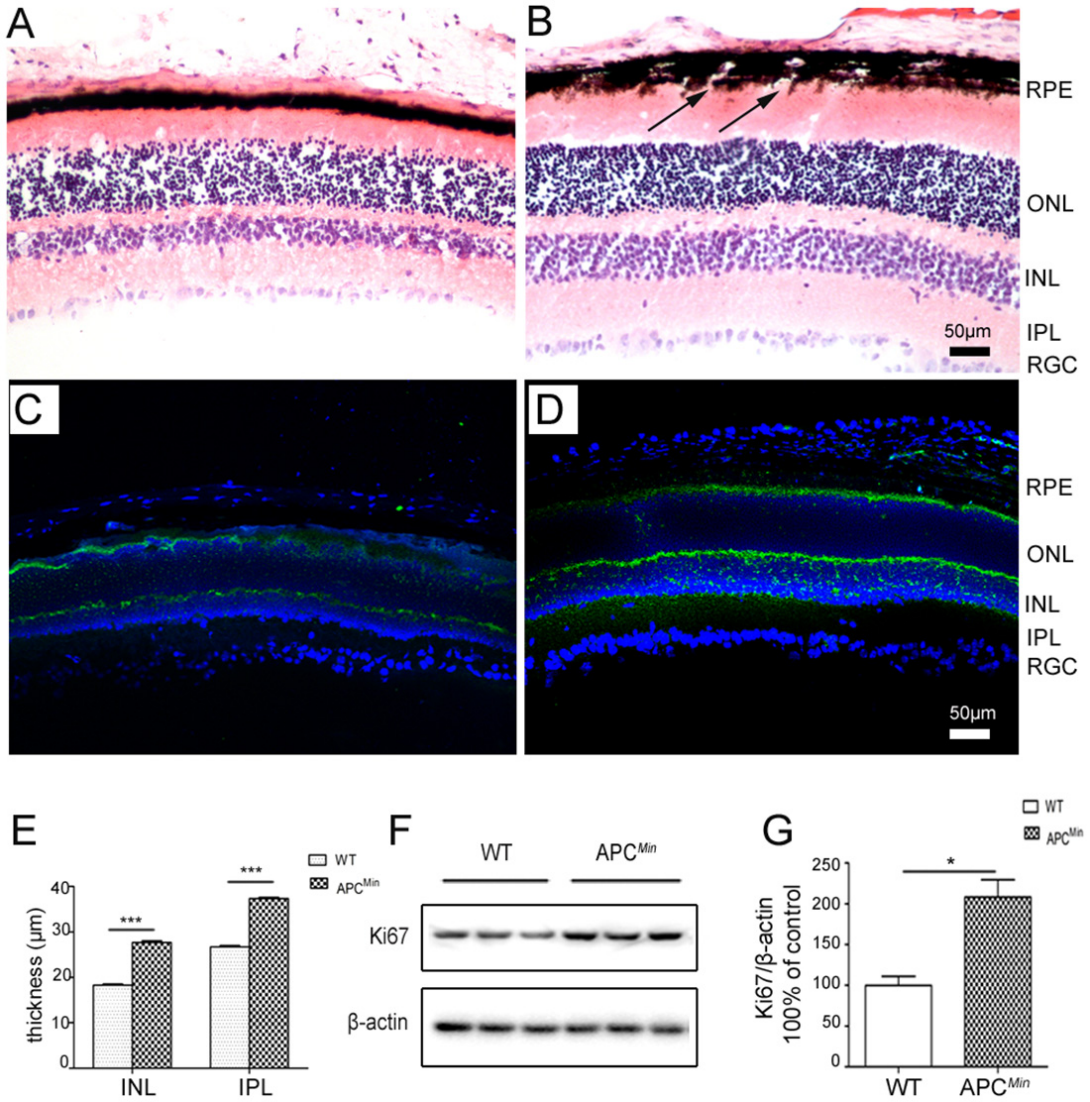
Comparison of retinal morphology at P84 in APC^min^ mice and age-matched WT littermates. H&E stained cross-sections show that the RPE membrane (arrows) is broken and the retina is thicker, especially in the INL and IPL (B) but not in age-matched WT control (A). Ki67 immunostaining of eye sections of WT (C) and APC^Min^ mice (D), The thickness of the INL and IPL was quantified in the APC^Min^ mice and WT mice (E). Fifty microgram of retinal proteins from P84 APC^Min^ mice and age-matched wild type (WT) mice was used for Western blot analysis of Ki67 (F), semiquantified by densitometry and normalized by β-actin levels (G) (mean ± S.D., n = 5), * *P*<0.05. RPE, retinal pigment epithelium; ONL, outer nuclear layer; INL, inner nuclear layer; IPL, inner plexiform layer; GCL, Ganglion cell layer.

During myopia development, retinal structural and functional changes occur [31,32]. Given the myopic shift in APC^Min^ mice, retinal histologic cross-sections were examined in APC^Min^ mice and age-matched WT mice at P84. Hematoxylin- eosin staining (H&E) staining showed an intact RPEmembrane in WT mice at P84 (Fig 3A). In contrast, RPE cell layer ruptures were evident in the APC^Min^ mice at the same time point (Fig 3B). Clinical studies also showed defects in the RPE membrane in the macular region of the highly axially myopic eyes [33].

Compared with age matched WT controls, the retina was significantly thicker in APC^Min^ mice at P84 (Fig 3A and 3B). On the other hand, there was no difference between APC^Min^ and age-matched WT in the outer nuclear layer (ONL) thickness. Nevertheless, inner nuclear layer (INL) and inner plexiform layer (IPL) of the APC^Min^ mice were thicker compared with those in WT mice (27.68±0.37 μm VS 18.27±0.29 μm, 37.36±0.37 μm VS 26.68±0.33 μm, APC^Min^ VS WT, *P*<0.001, n = 5, Fig 3E). To determine whether an increase in cell proliferation accounts for INL and IPL thickening, Ki67 expression levels were evaluated in western blots (n = 5). Previously, Geller et al. suggested that cellular Ki67 labeling (clone MIB-1) is a more accurate means of evaluating cellular proliferation in the retina and elsewhere in the CNS [34]. We found that Ki67 increased more in the APC^Min^ retinas than in age-matched WT mice (Fig 3C, 3D and 3F). Therefore, the current results provide evidence that the refractive error of the APC^Min^ mice is accompanied by increased retinal cell proliferation, which is consistent with a previous study [6].

### Tyrosine hydroxylase (TH) expression in APC^Min^ Mice

**Fig 4:**
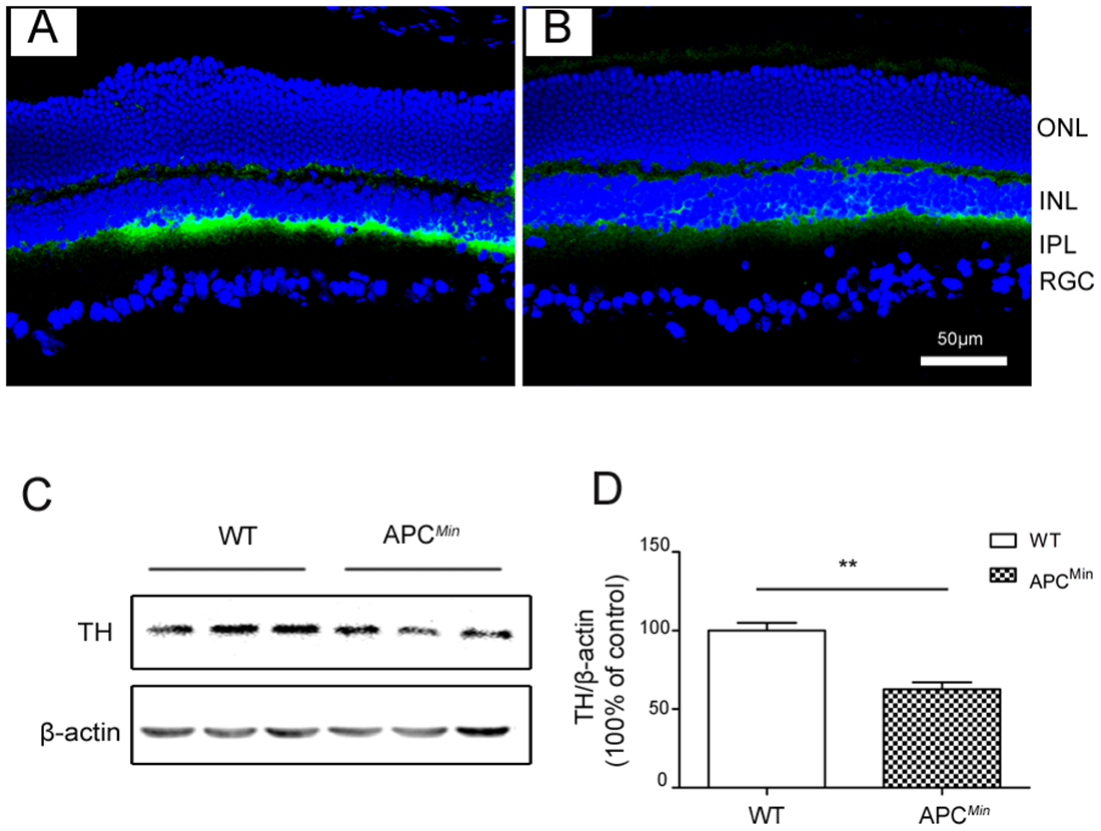
Diminished tyrosine hydroxylase (TH) retinal expression in P84 APC^Min^ mice. (Panels A and B). TH immunostaining of eye sections of WT (A) and APC^Min^ mice (B), showing decreased TH in the sub retinal space of APC^Min^ mice. The nucleus was counterstained with 4, 6-diamidino-2-phenylindole (DAPI) (blue). C, Fifty micrograms of retinal protein was used for western blot analysis of TH. D, Protein levels were semiquantified with densitometry, normalized by β-actin levels, and compared between APC^Min^ mice and WT mice littermates (mean ± SD, n = 5), ** *P*< 0.01.

DA has been implicated as a stop signal of ocular growth [14]. It is to be noted here that since retinal DA levels in the untreated control eye and FDM eye exhibit large inter-individual variability (more than 200%) [35], we therefore examined on P84 TH expression levels (the rate-limiting enzyme of DA synthesis [36]) to clarify if there is an association between between retinal DA levels and retinal proliferation in the APC^Min^ mice. Western blot and immunofluorescence analyses showed that TH levels, as compared with the WT retina, significantly decreased in the APC^Min^ mice (Fig 4). This decline suggests that in APC^Min^ mice down-regulated DA levels may enhance retinal proliferation, which has been previously reported [37].

### Association between myopia and posterior scleral collagen fibril diameter changes

**Fig 5:**
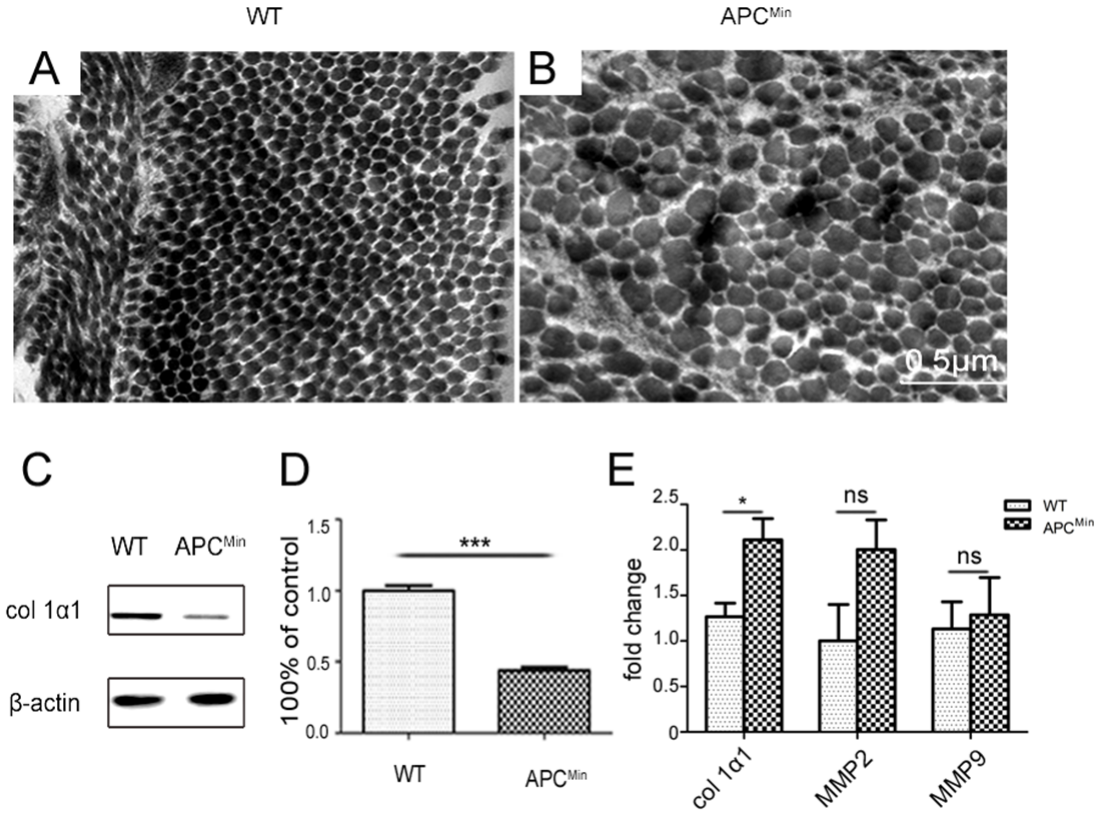
Scleral morphological changes in P84 APC^min^ mice and age-matched normal WT littermates. TEM compares collagen fibril morphology in cross-section from the posterior sclera of wild type (A) and APC^Min^ mice (B). A, WT fibrils have a regular, cylindrical contour whereas APC^Min^ mice sclera is more irregular and disorganized. The fibril contour diameters are variable having in some places a small diameter whereas in other locations it is larger (arrows). C, Col1α1 protein expression levels detected by western blot progressively decreased with development in APC^min^ scleras, as compared with WT mice littermates. D, Histogram illustrations of densitometry results of col1α1 protein expression levels. (Mean ± SD, n = 5), ** *P*< 0.01. E, real-time PCR measurements of col1α1, MMP2 and MMP9 in the retinas of APC^Min^ and WT mice. All mRNA levels are normalized to the control level of WT in P84 (mean ± SD, n = 5, *P < 0.05, **P < 0.01).

Scleral pathology underlies permanent declines in high myopes [38]. To clarify the possible structural basis for myopia development in APC^Min^ mice, we compared changes in their posterior scleral collagen diameter with those in WT littermates at P84. Ultrastructural analysis by electron microscopy revealed that the morphology of scleral collagen fibrils was dramatically altered in the APC^Min^ mice (Fig 5A and 5B). In the APC^Min^ mice, some areas had a higher density of small-diameter fibrils, whereas others had large-diameter irregular fibrils. This aberrant fibril structure is consistent with abnormal fusion of fibrils. To further determine the biochemical basis for these scleral changes, we compared col1α1 expression levels, since alterations in its expression are associated with myopia development [7]. Western blot analysis in APC^Min^ mice showed that scleral col1α1 expression declined, while its mRNA expression level increased compared to those in age matched WT mice (Fig 5C and 5D). These results suggest that in APC^Min^ mice altered col1α1 fiber metabolism may account for scleral disorganization. To determine whether the change in scleral col1α1 protein expression is accompanied by selective modulation of MMP2 and MMP9 mRNA gene expression, real-time PCR evaluated their scleral levels in APC^Min^ and WT mice. Analysis of MMP2 and MMP9 gene expression, normalized to the expression of the housekeeping gene β-actin, showed that there was no change in their levels in APC^Min^ mice, compared to those in the WT mice (Fig 5E). These results suggest that scleral col1α1 fiber downregulation in APC^Min^ mice is not attributable to any changes in MMP2 and MMP9 expression levels.

## Discussion

Myopia is a common cause of vision loss in a number of ocular disorders [39], but the mechanisms underlying its pathogenesis are not fully understood. It was reported that 10 out of 14 FAP patients with refractive error had myopia ranging from -0.5D to -10D [22]. The incomplete penetrance of myopia phenotype in these patients may be due to the differences in APC mutation sites, which has been shown to affect clinical manifestations [19]. In this study, we examined the alterations in the refractive and biometric parameters of APC^Min^ mice to elucidate the functions of APC during myopia progression.

To examine this issue, we used a custom-made real-time OCT and an eccentric infrared photo refractor (EIR) to compare refractive development in APC^Min^ mice with their age matched WT littermates. Such instrumentation designed by Xiangtian Zhou et al [29] allowed us to more accurately measure refractive status and other biometric parameters in mice. Our data suggest that APC expression is required for proper eye growth because APC^Min^ mice experience an extraordinary myopic shift and lenticular, retinal and scleral changes compared with age-matched WT littermates. Their development of this large myopic shift is consistent with abnormal eye size development. In contrast to invariant ACD and LT in human axial myopia [40], ACD became shallower and lenses shrunk in size in APC^Min^ mice relative to their age-matched WT littermates (Fig 2C and 2D). In addition, longer eye axis identified in a clinical setting is typically indicative of myopia [5]. Paradoxically, APC^Min^ mice instead usually have shortened- axial lengths and smaller eyes than their age-matched WT littermates (Fig 1C).

Myopia is a mismatch between the light-focusing power of the anterior segment and the axial length of the eye. As a result, the visual image comes to a focus in front of the retina [41]. Our results suggest that changes in thickness of lens and retina may not be able to compensate for the longer VC depth in later developmentwhich results in myopia in the APC^Min^ mice. Regardless of the underlying molecular mechanisms, the short-eyed myopia found in our study is different from that described in other gene-knockout myopia models [42–44]. Studies employing APC^Min^ mice may therefore provide novel insight into ocular development that is not readily accessible using instead other traditional models.

Developmental studies have revealed that proliferation of neurons in the marginal retina is highly correlated with the axial length of the eye during myopia development [6,45]. Accordingly, the addition of new neurons to the margin of the retina may compensate for retinal stretch that is imposed during myopia development [46]. Our analysis also indicated in P84 APC^Min^ mice that the retina was accompanied by more Ki67 expression than in normal eyes, which is indicative of rises in retinal layer cell proliferation and retinal thickness. The relationship between retinal thickness and myopia has been extensively investigated. In some reports, average macular thickness was invariant despite changes in myopia degree [47]. However, others found that in myopic eyes retinal thickness was greater at both its foveal center and in the rest of the fovea than that in the non-myopic group [48] [49]. On the other hand, the retina was instead significantly thinner in other zones of the macula in myopic eyes, compared with non-myopic eyes. Interestingly, we found that the retina in P84 APC^Min^ mice thickened. The exact mechanism underlying this enlargement needs further investigation. Thus, the APC gene mutation identified in FAP patients may contribute to increases in retinal cell proliferation.

There is emerging evidence suggesting that retinal DA level modulation affects eye growth [50]. Intravitreal injection of a dopaminergic neurotoxin, 6-hydroxydopamine, increased goldfish rod neuroblast proliferation [37]. Furthermore, in an APC mouse mutant model, the DA content is abnormally distributed in different brain regions, and these changes are associated with behavioral and phenotypic changes described in some neurological diseases [51]. In the context of the current study, it is conceivable that increases in retinal cell proliferation may be associated with decreased DA expression, which has been implicated as a ‘stop signal’ mediator of ocular growth [14]. To examine such a possibility, we measured changes in retinal TH expression, the rate-limiting enzyme of DA synthesis [52] and assumed they are reflective of variations in DA content. As the retinal TH protein expression level decreased in APC^Min^ mice (Fig 5), changes in APC gene expression may contribute to DA and TH expression regulation and retinal cell proliferation. Paradoxically, we found in APC^Min^ mice that the lenses were instead smaller than in their age-matched WT littermates. Additional studies are needed to clarify the underlying mechanism accounting for this response. Nevertheless, the current finding of a putative decline in retinal TH expression is consistent with previous indications that such an effect is associated with myopia development. Future studies are needed to determine whether DA receptor agonist and antagonist treatment change retinal proliferation and myopia development in the APC^Min^ mice. The molecular mechanism responsible for putatively downregulating DA expression in APC^Min^ mice requires future clarification.

Previous studies in humans and in myopia animal models suggest that scleral biology plays a pivotal role in eye size control and progression of this disease [53]. A possible function for the APC gene could include eye growth control by modulating scleral collagen expression. Our scleral ultrastructural analysis revealed regional heterogeneity in the fibril structures within delimited areas where there were either abnormally small- to very large-diameter fibrils. This result is consistent with findings in myopic tree shrews [54] and the Lum^-^/^-^Fmod^-^/^-^ double-null mouse [42]. Our western blot analysis in APC^Min^ mice showed that col1α1 decreased in scleral tissue (Fig 5C and 5D), while real-time PCR analysis showed no changes in MMP2 and MMP9 gene expression (Fig 5E) in APC^Min^ mice, compared to that in the WT mice. One explanation for the discrepancy between increased col1α1 degradation and invariant MMP2 and MMP9 gene expression is that other MMPs may be alternatively involved in cleaving type I collagen. Changes in col1α1 posttranscriptional regulation are another possibility to account for why its expression level declined. Taken together, these findings suggest that the effect of APC on the scleral changes is partially through regulation of col1α1 expression levels, which, in turn, could result in alterations in scleral architecture and severely affect vision.

APC suppresses Wnt signal transduction cascade by modulation the cellular expression levels of β-catenin [19]. Wnt signal is involved in the homeostasis of many tissues, such as intestine, skin, bone, eye and hematopoietic system In adults [55]. In addition, recent studies indicate the possible role of Wnt pathway in the development of myopia [56,57]. In this study, we found that APC^Min^ mice, in which Wnt signaling is activated, displayed myopic shift. Therefore, it provides additional evidence for the role of the Wnt signal pathway in the myopia development. Furthermore, previous study reported that stimulation of the Wnt pathway through inhibition of GSK-3β, a key enzyme in Wnt signal pathway that destabilizes β-catenin, increased retinal Ki67 expression. [58]. The thickened retina and increased Ki67 in the APC^Min^ mice in our study suggest that the Wnt signal pathway may be involved in the control of proliferation of retina, which needs further study.

Previous study found that the APC gene mutation causes significant histological abnormalities in several proliferative tissues [59]. Similarly, our results in Table 1 found that the ocular biometric parameters in APC^Min^ mice proportionally grow larger than in WT animals, whereas changes in body weight increase less over time than in WT mice. Our finding is consistent with a previous study that the APC gene mutation has apparently different effect in different tissues [59][59]. The mechanism and signaling pathways responsible for the observed changes remain to be elucidated in the future.

Our study has the following limitations. 1), it is not clear whether the ocular and refractive changes in the APC^Min^ mice are the same as those occurring in other mutation sites. 2), we did not evaluate whether the ocular biometric parameter changes accompanying myopia development in APC^Min^ mice also appear in human subjects with APC mutations, which could be of interest in further investigations.

In summary, this study describes for the first time refractive development in APC^Min^ mice. The results suggest that APC expression in mice is essential for post natal development of error-free refraction based on measurements of time dependent changes in LT, VCD, and AL. Furthermore, our findings prompt additional genetic studies on APC polymorphism and its related signaling pathways as they may be related to myopia development.

## Supporting Information

S1 ARRIVE Checklist ARRIVE Guidelines Checklist. Animal Research: Reporting In Vivo Experiments.
(PDF)
